# Impact of sex and age on vaccine-related side effects and their progression after booster mRNA COVID-19 vaccine

**DOI:** 10.1038/s41598-023-46823-4

**Published:** 2023-11-07

**Authors:** Masahiko Mori, Aiko Yokoyama, Ayami Shichida, Kimiko Sasuga, Takafumi Maekawa, Tadayoshi Moriyama

**Affiliations:** 1 Department of Internal Medicine, Sasebo Memorial Hospital, Sasebo, Nagasaki 858-0922 Japan; 2 Regional medical cooperation office, Sasebo Memorial Hospital, Sasebo, Nagasaki 858-0922 Japan; 3 Medical Administration Division, Sasebo Memorial Hospital, Sasebo, Nagasaki 858-0922 Japan; 4 Department of Medical Information, Sasebo Memorial Hospital, Sasebo, Nagasaki 858-0922 Japan; 5 Department of Surgery, Sasebo Memorial Hospital, Sasebo, Nagasaki 858-0922 Japan; 6 Department of Surgery, Fukuoka Central Hospital, Fukuoka, Fukuoka 810-0022 Japan; 7 Department of Neurosurgery, Sasebo Memorial Hospital, Sasebo, Nagasaki 858-0922 Japan

**Keywords:** Viral infection, RNA vaccines, Drug safety

## Abstract

In mRNA COVID-19 vaccination, side effects after the first and second dose have been well reported. However, studies about side effects after booster vaccine are sparse. 272 healthcare workers who received the third mRNA COVID-19 vaccine were recruited, and impact of sex, age, and symptoms on the side effect progression was statistically analyzed. Females and younger adults had a higher frequencies of general fatigue, headache, joint pain, chills and axillary pain compared to males and elderly adults, respectively. In longitudinal analysis, prolonged time to recovery from side effects was found among females and younger adults. Finally, between the third and second dose vaccinations, 52% of subjects had a longer duration of side effects following the third vaccine compared to the second, and joint pain was the culprit symptom related to the prolonged duration of side effects. Following the second vaccine dose, 25% of subjects had a longer duration of side effects and asthma and ear fullness, which exacerbated the underlying allergic condition, and COVID arm symptom were the culprit symptoms. These highlight the impact of sex, age, and culprit symptoms on the progress of side effects following the booster mRNA COVID-19 vaccine.

## Introduction

Administration of the newly developed mRNA COVID-19 vaccines was initiated in Japan in February 2021, with priority given to the healthcare personnel. The BNT162b2 mRNA vaccine (Comirnaty®) (Pfizer, New York, NY, USA, and BIONTECH, Mainz, Land Rheinland-Pfalz, Germany) was used initially, followed by mRNA-1273 vaccine (COVID-19 vaccine Moderna®) (Moderna, Cambridge, MA, USA). By April 2023, nearly 104 million people in Japan (81% of population) received their first dose, and 103 million people (80% of population) received their second dose^1^. However, despite its clinical and immunological benefits^2–4^, only 86 million people (68% of population) received the third vaccine dose.

One of the reasons for vaccine hesitation is concern about side effects^5^. Following the first two vaccine doses, multiple studies published data on vaccine-related side effects, including data regarding frequency of acute and severe side effects, such as anaphylactic shock^6,7^, and swelling or bleeding at the injection site^8–10^. In a previous cohort study after the first and second doses of COVID-19 vaccines, various host and vaccine factors such as sex, age, vaccine brand, race, pre-existing conditions of asthma and anemia, marijuana use, pregnancy at baseline, and subjective social status were identified as significant factors associated with vaccine-related side effects^11^. In terms of age-related side effects, higher frequencies of local and systemic side effects among younger adults (age ≤ 64 years old) compared to elderly adults (age ≥ 65 years old) were previously reported^12,13^. On the other hand, information about vaccine-related side effects after the third vaccine dose and how they compare to the first and second vaccine doses remains sparse and incomplete. Our objective here was to identify the impact of sex and age on vaccine-related side effects and their progression after the third dose mRNA COVID-19 vaccine in a cohort of 272 vaccinated adults from Japan.

## Results

### Characteristics of the cohort

Of the 272 vaccinated individuals, 216 (79%) were female and 56 (21%) were male (Table 1). Median age at enrollment was 47 years (interquartile range (IQR) 36–57). Median body temperature prior to vaccination was 36.4℃ (IQR 36.2–36.6). In terms of the immunologic background, 41 (15%) had past history of immune response-related diagnosis or event. Of these, 26 (9.6%) had an allergy diagnosis, 12 (4.4%) had collagen disease diagnosis, and 10 (2.9%) had a history of side effects from previous vaccinations. Seven (2.6%) had a history of seizures.

**Table 1:** Characteristics of the cohort.

Characteristic (n = 272)	Number (%)
Sex (female)	216 (79)
Age	^a^47 (36–57)
Body temperature (℃)	^a^36.4 (36.2–36.6)
Immunologic history	41 (15)
Allergy diagnosis	26 (9.6)
Collagen disease diagnosis	12 (4.4)
Side effects from prior vaccination	10 (2.9)
Seizure history	7 (2.6)

^a^Median (interquartile range) is shown.

### Higher number of vaccine-related side effects among females

We first investigated whether the number of side effects was significantly different between females and males following the third vaccine dose. In total, 36 symptoms were identified, and females experienced a significantly higher number of side effects compared to males: median 6 vs 3, respectively (p < 0.001) (Table 2). This statistical significance persisted for each individual symptom tested, including general fatigue (odds ratio (OR) 2.6, p = 0.002, q = 0.032), headache (OR 3.4, p < 0.001, q = 0.004), swelling at the injection site (OR 2.3, p = 0.006, q = 0.053), joint pain (OR 2.3, p = 0.010, q = 0.078), chills (OR 2.7, p = 0.004, q = 0.043), and tendency in axillary pain (OR 3.8, p = 0.021, q = 0.130) (Table 2 and Supplementary Table 1). These data support the notion of sex differences in developing COVID-19 vaccine-related adverse symptoms.

**Table 2:** Sex and age differences in vaccine-related side effects.

Symptom		+	–	(%)	^a^OR	p	q
Sex							
^b^Number of symptoms	Female	6 (3–7)				< 0.001	
	Male	3 (2–5)					
General fatigue	Female	150	66	(69)	2.6	0.002	0.032
	Male	26	30	(46)			
Headache	Female	145	71	(67)	3.4	< 0.001	0.004
	Male	21	35	(38)			
Swelling at the injection site	Female	141	75	(65)	2.3	0.006	0.053
	Male	25	31	(45)			
Joint pain	Female	103	113	(48)	2.3	0.010	0.078
	Male	16	40	(29)			
Chills	Female	97	119	(45)	2.7	0.004	0.043
	Male	13	43	(23)			
Axillary pain	Female	38	178	(18)	3.8	0.021	0.130
	Male	3	53	(5.4)			
Age							
^b^Number of symptoms	≤ 64	6 (3–7)				< 0.001	
	≥ 65	2 (1–3)					
General fatigue	≤ 64	169	78	(68)	5.6	< 0.001	0.002
	≥ 65	7	18	(28)			
Headache	≤ 64	160	87	(65)	5.8	< 0.001	0.002
	≥ 65	6	19	(24)			
Joint pain	≤ 64	118	129	(48)	22	< 0.001	< 0.001
	≥ 65	1	24	(4.0)			
Chills	≤ 64	107	140	(43)	5.6	0.002	0.021
	≥ 65	3	22	(12)			
Fever (≥ 37.5 ℃)	≤ 64	86	161	(35)	6.1	0.006	0.045
	≥ 65	2	23	(8.0)			
Axillary pain	≤ 64	41	206	(17)	-	0.019	0.118
	≥ 65	0	25	(0)			

Symptoms with p < 0.05 by Fisher’s exact test in frequency of vaccine-related side effects and their q values by false discovery rate analysis are shown. Mann–Whitney *U*-test is applied for the analysis of differences in the number of symptoms. Results for all side effects are shown in Supplementary Table 1 and Supplementary Table 2.

^a^*OR* Odds ratio.

^b^Median (interquartile range) is shown.

“ + ” indicates presence of a symptom.

“–” indicates absence of a symptom.

### Higher number of vaccine-related side effects among younger adults

To investigate the effect of age on vaccine-related side effects, we next analyzed frequency of side effects in younger adults (≤ 64 years old) versus elderly adults (≥ 65 years old). There was a higher number of side effects in younger adults compared to elderly adults: median 6 vs 2, respectively (p < 0.001) (Table 2). This statistical significance persisted for each individual symptom, including general fatigue (OR 5.6, p < 0.001, q = 0.002), headache (OR 5.8, p < 0.001, q = 0.002), joint pain (OR 22, p < 0.001, q < 0.001), chills (OR 5.6, p = 0.002, q = 0.021), fever (OR 6.1, p = 0.006, q = 0.045) and tendency in axillary pain (OR -, p = 0.019, q = 0.118) (Table 2 and Supplementary Table 2). These results suggest that age is another important factor contributing to the COVID-19 mRNA vaccine-related side effects.

### Multivariable analysis: sex and age remained independent factors influencing the number of vaccine-related side effects

To confirm the impact of sex and age on vaccine-related side effects, we used multivariable analysis (Table 3). Both sex and age remained significant and independent factors affecting the number of vaccine-related side effects, as follows: for sex (females compared to males), B = 1.7, 95% confidence interval range (CI) 0.9–2.5, p < 0.001; for age, B = − 0.04 (95% CI − 0.06 to − 0.01), p = 0.003. These results support the significant and independent effects of sex and age on the number of COVID-19 vaccine-related side effects.

**Table 3:** Impact of characteristics of the cohort on the number of vaccine-related side effects.

	Univariable		Multivariable	
	B (95% ^a^CI)	p	B (95% ^a^CI)	p
Sex (female)	1.8 (0.9–2.6)	< 0.001	1.7 (0.9–2.5)	< 0.001
Age	− 0.04 (− 0.07– − 0.02)	< 0.001	− 0.04 (− 0.06– − 0.01)	0.003
Body temperature (°C)	1.1 (0.06–2.1)	0.038	0.4 (− 0.6–1.4)	0.420
Immunologic history	− 0.01 (− 0.9–0.9)	0.984		
Allergy diagnosis	0.3 (− 0.9–1.4)	0.665		
Collagen disease diagnosis	− 1.1 (− 2.8–0.5)	0.182		
Side effects from prior vaccination	0.4 (− 1.7–2.4)	0.728		
Seizure history	− 0.7 (− 2.9–1.4)	0.494		

Linear regression model analyses are shown. Variables with significance (p < 0.05) in univariable analysis were applied to the multivariable analysis.

^a^*CI* confidence interval range.

### Longitudinal analysis: prolonged recovery rate from vaccine-related side effects in females and younger adults

**Figure 1:**
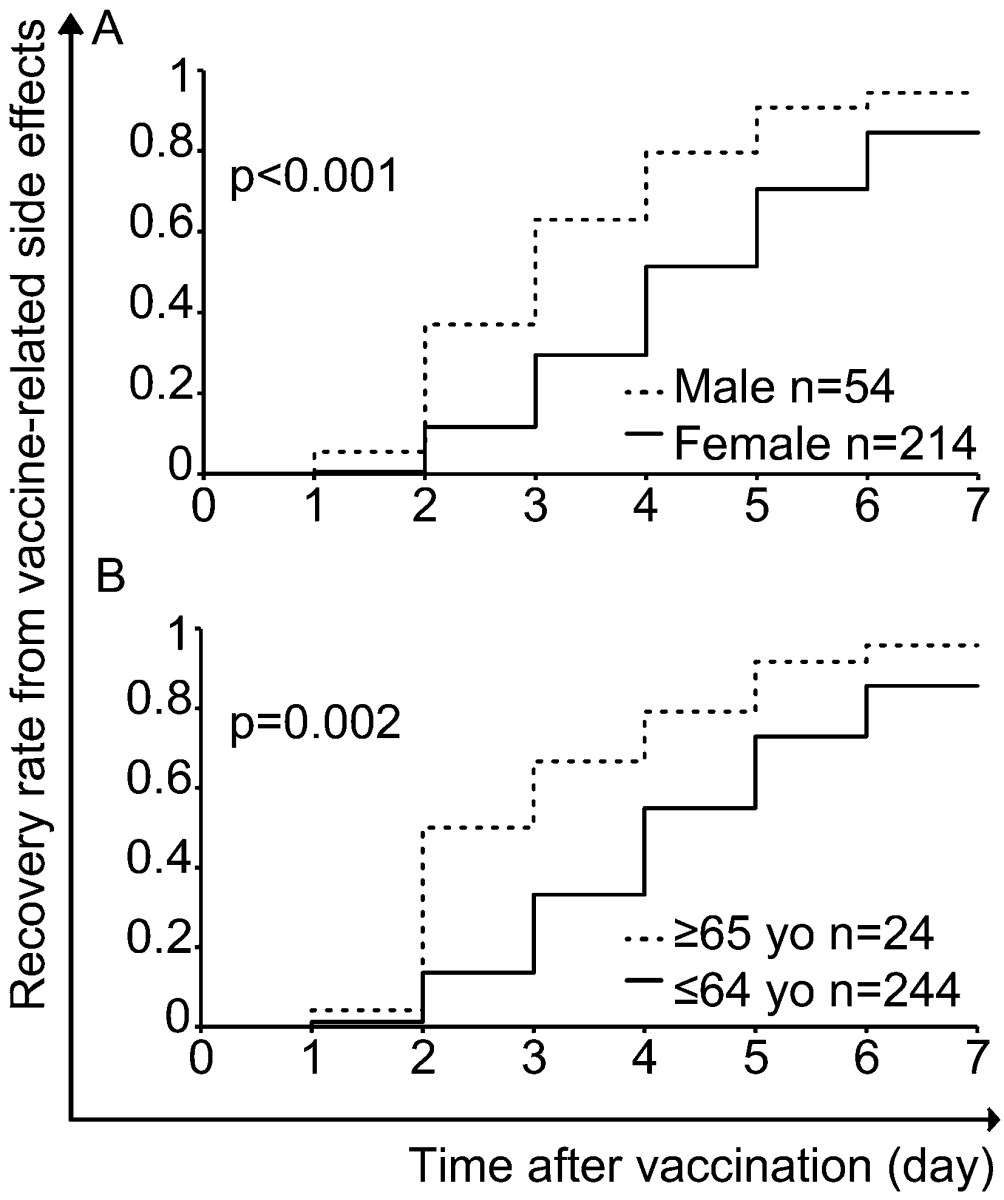
Sex and age differences in recovery rate from side effects, after the third vaccine dose. Log-rank tests are shown.

Next, we assessed the impact of sex and age on the recovery rate from vaccine-related side effects among 268 out of 272 subjects who had adverse symptoms following third vaccine dose. The duration until full recovery from all vaccine-related side effects after vaccination was a median of 4 (IQR 3–6) days in females vs 3 (2–4) days in males (p < 0.001); and 4 (3–6) days in younger adults vs 2.5 (2–4) days in elderly adults (p < 0.001). We found that the recovery rate from a side effect was significantly prolonged among females compared to males, and among younger adults compared to elderly adults: 85% vs 94% recovery rate in females vs males, respectively, p < 0.001 (Fig. 1A) and 86% vs 96% in younger vs elderly adults, respectively, p = 0.002 (Fig. 1B). These findings indicate sex and age differences in the rate of recovery from vaccine-related side effects.

### Discrepancy in the duration of side effects following the third vs second vaccine doses

**Figure 2:**
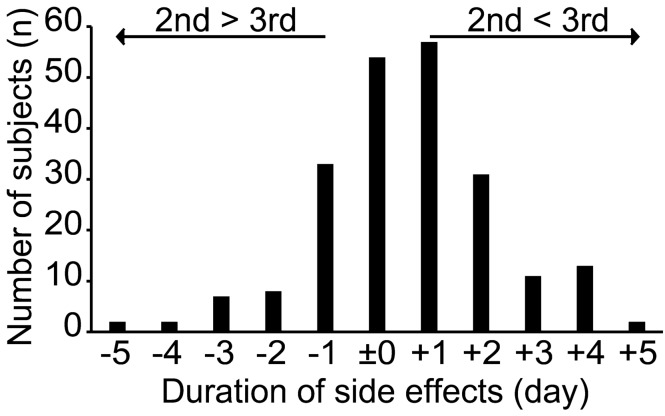
Differences in the duration of side effects following the third vs second vaccine doses. Distribution of difference in the duration of side effects after the third vaccine and second vaccine dose is shown. “ + ” indicates the increase in duration of side effects after the third vaccine dose compared to the second vaccine dose. “–” indicates the decrease in duration of side effects after the third vaccine dose compared to the second vaccine dose.

Some, but not all patients reported that they experienced more severe side effects following the third vaccine dose compared to those following the second vaccine dose. To address this, we compared the duration of the side effects following the third vs second vaccine doses in each patient. Additionally, we analyzed the role of each individual side effect on the duration of symptoms after each vaccine dose. Two hundred and twenty subjects had information regarding their side effects following both the third and second vaccine doses. We found that 114 (52%) subjects had a longer duration of side effects after the third vaccine dose compared to the second vaccine dose, and 52 (24%) had a longer duration of side effects after the second vaccine dose compared to the third vaccine dose (Fig. 2). Linear regression model analysis demonstrated that joint pain after the third vaccine dose was the symptom significantly contributing to the longer duration of side effects following the third vaccine dose (B = 0.8, p = 0.002) (Table 4 and Supplementary Table 3). On the other hand, asthma (B = − 3.4, p = 0.042), ear fullness (B = − 4.3, p = 0.019), and bleeding at the injection site (B = − 3.2, p = 0.007) were identified to be significant symptoms contributing to the longer duration of side effects following the second vaccine dose.

**Table 4:** Symptoms effecting on side effect duration discrepancy between the third and second vaccine dose.

	Univariable		Multivariable	
	B (95% ^a^CI)	p	B (95% ^a^CI)	p
Sex (female)	0.5 (− 0.3–0.7)	0.079		
Age	0.008 (− 0.009–0.026)	0.359		
3rd dose vaccine				
Headache	0.5 (0.05–1.0)	0.031	0.2 (− 0.3–0.6)	0.529
Joint pain	0.9 (0.4–1.3)	< 0.001	0.8 (0.3–1.2)	0.002
2nd dose vaccine				
Asthma symptom	− 3.6 (− 7.0– − 0.1)	0.041	− 3.4 (− 6.6– − 0.1)	0.042
Ear fullness	− 5.6 (− 9.0– − 2.2)	0.001	− 4.3 (− 7.8– − 0.7)	0.019
Numbness at upper arm	− 1.6 (− 3.0– − 0.2)	0.026	− 1.0 (− 2.4–0.5)	0.190
Bleeding at the injecting site	− 3.1 (− 5.5– − 0.7)	0.013	− 3.2 (− 5.5– − 0.9)	0.007

Linear regression model analyses are shown. + in B indicates the increase of side effect duration after the third vaccine dose compared to the second vaccine dose, and – indicates the decrease of the duration after the third vaccine dose compared to the second vaccine dose. Variables with significance (p < 0.05) in univariable analysis were applied to the multivariable analysis. Results for all side effects are shown in Supplementary Table 3.

^a^*CI* confidence interval range.

## Discussion

This study systematically investigated the impact of sex and age on the side effects following the third vaccine dose in a cohort of COVID-19 mRNA vaccinated adults. Here, using cross-sectional and longitudinal analyses, we identified significantly higher frequency of several vaccine-related adverse symptoms and prolonged recovery rate in females and younger adults following the third vs second vaccine doses. Additionally, we found several individual side effects that significantly contributed to the duration of side effects.

Our findings of higher frequencies of COVID-19 vaccine-related side effects among females and younger adults compared to males and elderly adults were consistent with previous reports^6,7,11–13^. Additionally, our longitudinal analyses revealed worse outcomes with longer recovery from side effects in females and younger adults compared to those in males and elderly adults. Other studies reported sex differences in vaccine response and higher vaccine efficacy but worse adverse reactions in females vs males, including vaccines against influenza, hepatitis B and yellow fever^14–16^. Higher number of B cells resulting in greater antibody production in females^17^ and increased stimulation of immune cells by female sex hormones (estrogen, progesterone), as well as suppression by male sex hormones (testosterone) may be considered a plausible mechanism of sex differences in responses to vaccines^18,19^.

In terms of adverse age-related impact on the frequency and duration of side effects following vaccine, decline in immune function with age, referred to as immunosenescence, should be considered^20,21^. Effect of immunosenescence on decline of vaccine efficacy was reported with other vaccines such as influenza, varicella zoster, and the combination vaccine against tetanus, diphtheria, and pertussis^22–24^. Our findings of higher number and longer duration of side effects among younger adults support these data.

Axillary pain is a side effect that occurred at a significantly higher frequency following the third vaccine dose compared to the first or second vaccine doses. After the first and second doses in the BNT162b2 trial, axillary swelling was recorded as an unsolicited reaction only^25^. In the mRNA-1273 trial, axillary swelling and tenderness were reported in 11.6% patients after the first vaccine dose and in 16% after the second vaccine dose^26^. In our previous study of BNT162b2 and related side effects, frequency of axillary lymph node swelling or axillary pain was 0% (0/262) after the first vaccine dose, 3.9% (10/257) of lymph node swelling after the second vaccine dose^27^, and 15% (41/272) of axillary pain in this third vaccine dose study. Ipsilateral vaccine-related reactive axillary lymphadenopathy was demonstrated in multiple radiologic studies, such as screening mammograms^28–30^ or cancer surveillance PET CT studies. Asymmetric axillary lymphadenopathy is a concerning imaging finding for radiologists since the differential diagnosis includes nodal metastatic disease^31^. These notions underscore the importance of obtaining COVID-19 vaccination history prior to image examinations.

Several symptoms were identified as the culprit symptoms contributing to the prolonged duration of side effects following the third vs second vaccine doses, specifically joint pain after the third vaccine dose, and asthma, ear fullness, and bleeding at the injection site after the second vaccine dose. A possible mechanism could be related to previous studies of mRNA COVID-19 vaccine^32^ and influenza vaccines^33–35^, which reported an increase of proinflammatory cytokines such as TNF-α and IL-6, and a decrease of extracellular vesicle immune-regulatory microRNA levels following vaccination. Levels of these proinflammatory cytokines and extracellular vesicle microRNA may stimulate systemic side effects following the third vaccine dose mRNA vaccine, which we describe in this study.

On the other hand, asthma and ear fullness after the second vaccine dose were identified as significant symptoms prolonging the duration of side effects after the second vs third vaccine doses. Notably, asthma and ear fullness are allergic symptoms which had already been present in the individuals prior to vaccination and were exacerbated by the vaccine. Previously reported systemic immune response syndrome (SIRS)^36,37^ and its association with upregulation of genes involved in neutrophil degranulation and cytokine signaling^38^ may be considered as a potential mechanism of our findings. These notions underscore the importance of obtaining a thorough history about an individual’s past medical diagnoses or treatments prior to vaccination.

Bleeding at the injection site after the second vaccine dose was also identified as one of culprit symptoms prolonging the duration of side effects following the second vaccine dose. This local dermatological symptom is known as ‘COVID arm’^8,27^. Delayed hypersensitivity reaction by type IV allergic response was proposed as the mechanism^9,10^.

Our findings about the different individual symptoms affecting the duration and severity of the vaccine-related side effects suggest that immune responses that generate the side effects differ between the third vaccine dose (systemic inflammation) and second vaccine dose (type I and IV allergic responses). Further immunological studies including cytokine and antibody level measurements would be warranted, and these findings would contribute for the understanding of mechanism of mRNA vaccine-related side effects.

As a limitation of this study, a small number of subjects for the vaccine-related side effect study, with discrepancies in sex and age distribution, were considered. Since the subjects in this study were derived from healthcare workers, distribution discrepancies with more females and younger adults occurred. For further analyses, a larger number of subjects with an equal distribution of sex and age should be considered. With regard to the subjects, while all of the subjects in this study had no history of COVID-19 diagnosis prior to vaccination, the inclusion of asymptomatic cases among them was considered another limitation of this study. In a study involving the Japanese population, the frequency of asymptomatic cases was 0.33% out of one million tested individuals in 2021^39^, and 1.1% (23 out of 2185) among healthcare workers^40^. Considering the higher frequency of vaccine-related side effects among subjects with a past history of COVID-19 infection compared to those without such a history^41,42^, the detection and exclusion of asymptomatic cases through anti-COVID-19 IgG measurement would be warranted for further analyses.

In conclusion, this study investigated the impact of sex and age on mRNA COVID-19 vaccine-related side effects in booster-vaccinated adults (i.e. adults who received the third vaccine dose). We found that vaccine-related side effects are more frequent among females and younger adults, and that these two groups have a prolonged recovery compared to males and elderly adults. We also identified the individual culprit side effects that influence the duration of vaccine-related adverse effects following the third vs second dose. Specifically, we identified the significant negative contribution of systemic symptoms such as joint pain and headache after the third vaccine dose, and exacerbation of an underlying allergic condition and type IV allergic response after the second vaccine dose. Identification of the unique sex- and age-specific adverse symptoms, as well specific side effects characteristic of third and second COVID-19 vaccine doses will provide an opportunity to better understand the nature of sex- and age-associated immunological differences and develop safer and more efficacious vaccines.

## Methods

### Subjects and data collection

This research was approved by the ethical review board at Sasebo Memorial Hospital, Japan (approval number 2022-1). This cohort study was originally approved and initiated for research on COVID-19 vaccine-related side effects after the first and second doses (approval number 202107)^27^, and it has been continuously approved for this study. The research associated with human data use has complied with all the relevant national regulations and institutional policies, and was conducted in accordance with the tenets of the Helsinki Declaration. All participants provided written informed consent for the collection of information about side effects and subsequent analysis. A total of 272 hospital employees who received BNT162b2 COVID-19 vaccine (Cominarty®) (Pfizer, New York, NY, USA, and BIONTECH, Mainz, Land Rheinland-Pfalz, Germany) as the third vaccine dose were recruited from January to June 2022. Two hundred twenty of 272 subjects also had side effect information following the second vaccine dose, and this data was used for comparative analyses between the second and the third vaccine doses. None of the study participants had history of COVID-19 diagnosis prior to vaccination. Background information, such as body temperature prior to vaccination, past immunologic history (for example, diagnosis of allergy or collagen disease or side effects from prior vaccination), and seizure history, was collected using the national Pre-vaccination Screening Questionnaire for COVID-19 vaccine form, issued by the Japanese Ministry of Health, Labor and Welfare (Supplementary Fig. 1). Symptoms after vaccination was interviewed, diagnosed and collected by medical doctors at outpatient of the hospital studied.

### Statistical analysis

Statistical analysis was performed using SPSS® 21.0 (IBM, Armonk, NY, USA). The effect of sex (female vs male) and age (younger adults (≤ 64 years old) vs elderly adults (≥ 65 years old)) on the number of side effects was tested by Mann–Whitney U-test, with confirmation of non-normal distribution in each group by Shapiro–Wilk test. Referring to previous reports on the differences in side effects between these younger and elderly adult groups after the first and second vaccine doses^12,13^, we included these groups in this study. Sex and age differences in frequency of clinical symptoms were analyzed by Fisher’s exact test with false discovery rate analysis. Linear regression model with multivariable analysis was used to evaluate the impact of sex and age on the number of side effects. In longitudinal analysis, log-rank test was performed to assess sex and age differences in recovery rate from the vaccine-related side effects. Finally, linear regression model was applied to evaluate the impact of the culprit side effect symptoms on the difference in side effect duration between the second and third vaccine doses.

### Supplementary Information

Supplementary Figure 1.

Supplementary Table 1.

Supplementary Table 2.

Supplementary Table 3.

## Data Availability

The datasets generated during and/or analyzed during the current study are available from the corresponding author on reasonable request.
